# Impact of long duty hours on education and well-being of diagnostic radiology residents: A national survey in Saudi Arabia

**DOI:** 10.12669/pjms.38.3.4440

**Published:** 2022

**Authors:** Ayman S Alhasan, Shahad M Alahmadi, Yara A Altayeb, Tareef S Daqqaq

**Affiliations:** 1 Ayman S Alhasan, MBBS, DES. Department of Radiology, Assistant Professor and Consultant Radiologist, College of Medicine, Taibah University, Madinah, Saudi Arabia; 2 Shahad M Alahmadi, MBBS. College of Medicine, Taibah University, Madinah, Saudi Arabia; 3 Yara A Altayeb, MBBS. College of Medicine, Taibah University, Madinah, Saudi Arabia; 4 Tareef S Daqqaq, MBBS, Facharzt Department of Radiology, Associate Professor and Consultant Radiologist, College of Medicine, Taibah University, Madinah, Saudi Arabia

**Keywords:** Long Duty Hours, Radiology, Residents, Saudi Arabia, Well-being

## Abstract

**Objectives:**

The primary purpose of this study was to assess and report the perceived negative impact of long duty hours on education and personal well-being among medical trainees in the diagnostic radiology residency training program in Saudi Arabia.

**Methods:**

This cross-sectional study used a questionnaire (sent by email) with eight indicators related to the education and well-being of radiology residents in Saudi Arabia during the academic year 2019–2020. Participants were given a five-point Likert response format for each indicator. The relative importance index (RII) was calculated to rank the different indicators.

**Results:**

Our of 337 residents, 116 diagnostic radiology trainees completed the survey, with a response rate of 34.4%. A total of 102 (87.9%) indicated their preference for 16-hour shifts instead of the currently implemented 24-hour duty system. Using the RII, three items related to the post-duty day ranked at the top of the list. The negative impact on sleep rhythm during the post-call day ranked first (mean 4.23 ± 1.02, RII 0.84), followed by the impact on social life, family activities, and exercise during the post-call day (mean 4.09 ± 1.06, RII 0.81). The third highest ranking factor was missing academic activities on the post-call day (mean 3.91 ± 1.15, RII 0.78). There was no relationship between negative perception and gender (*P* > 0.05).

**Conclusion:**

The 24-hour duty system had a negative impact on radiology residents’ education and personal well-being, especially for items related to the post-call day. Reforming duty hours should be considered to promote residents’ well-being.

## INTRODUCTION

Medical residency after graduation from medical school is a stressful period of acquiring occupational skills that prepare physicians to enter into independent practice.^1^ Radiology residency training programs in the Kingdom of Saudi Arabia have undergone substantial expansion and evolution over the last decade under the supervision of the Saudi Commission for Health Specialties (SCFHS).^2^ Despite different studies showing the detrimental effects of long duty hours on residents in training,^3^,^4^ the long-standing tradition of 24-hour duty is still widely implemented in most radiology residency training programs in Saudi Arabia. In this system, after eight hours of regular work, the on-call resident continues with on-call duty, which typically starts at 4 or 5 p.m. and finishes at 8 a.m. the next day. Most clinical care decisions are based on the residents’ preliminary readings during on-call duty. According to the SCFHS regulations, continuation of daily duties post-call is encouraged to enhance clinical exposure, but not mandatory, as the SCFHS recommends a maximum of 24 hours of in-hospital duty hours.^2^

There is conflict in the literature about long working days that require the residents to participate on-call for 24 hours. Several studies have shown that long duty hours negatively affect physicians’ well-being and quality of life, altering mood and sleep, affecting memory, increasing burnout, impairing cognitive and clinical performance, and increasing the chances of medical error.^3^-^5^ However, other studies claim that on-call shifts of 24 hours are beneficial in medical residents’ training and do not negatively affect patient safety.^6^,^7^

Both the physiological and emotional effects and the impact on performance are different perspectives that have been studied among healthcare professionals on 24-hour on-call shifts. Sleep deprivation is the major outcome that causes an increase in stress hormone levels and blood pressure, a decrease in parasympathetic tone, and other general symptoms such as a deterioration in alertness, attention and general executive functions.^8^ When studying the performance of healthcare workers after on-call shifts of 24 hours, studies have mostly focused on the performance of cognitive tasks, monitoring tasks and simulated clinical tasks.^9^,^10^ Furthermore, various studies have reported burnout in radiology residents or trainees due to long working hours.^11^,^13^ It is difficult to make decisions related to duty hour restrictions based on the above-mentioned studies given the substantial variation between Saudi Arabia and other countries in terms of the postgraduate medical education, the infrastructure of the health system, and the local culture.

The impact of long duty hours on radiology residents’ education and well-being in Saudi Arabia is still unclear. Advocates for duty hour restriction are calling for immediate change based on studies performed on residents from other countries, while opponents draw different conclusions. To the best of our knowledge, a national survey of radiology residents has not been undertaken. The aim of this study was to investigate and assess the negative impact of the currently implemented 24-hour work system on radiology residents’ education and well-being in Saudi Arabia.

## METHODS

This is a cross-sectional study, including any radiology resident enrolled at any level of the residency program in Saudi Arabia during the 2019–2020 academic year. Radiology residents not registered in the residency program in September 2020, registrars, and fellows were excluded. The survey was open between September 15, 2020, and October 14, 2020. Responses were collected electronically with no identifying information. Since our population was easily accessible through the e-mail group of the SCFHS, the anonymous web-based survey was distributed electronically by e-mail to all eligible radiology residents in the Kingdom of Saudi Arabia. The link to the survey with a personalized message was also circulated through WhatsApp (Facebook, Menlo Park, CA, USA) Group of residents-in-training within the Kingdome of Saudi Arabia. The regional and local training program directors were asked to encourage residents’ participation. The study protocol and the survey instrument were reviewed and received ethical approval from the Institutional Review Board (Ethical approval number 004-1442).

Participants were informed that their participation is anonymous and their responses are confidential. An online survey development tool (Google; Mountain view, California, USA) was used to collect the responses. The items and questions in the survey were developed by a multidisciplinary work group after a review of previous studies in the literature.^14^ The items in the survey were prepared and refined after discussion with a selected group of residents in the training program to allow better understanding of the indicators related to the negative impact of long duty hours.

### The survey contained three parts:

- The first part was dedicated to collecting demographic data, including age, gender, level, and administrative region of training.
- The second part included seven questions. Two questions aimed to assess and evaluate the perceived negative impact during night duty on i) overall performance; and ii) decision-making capability. Four questions aimed to assess the negative impact post-duty on i) academic activities; ii) social life, family activities, and exercise; iii) mood and humor; and iv) sleep rhythm. The last question aimed to assess the overall negative impact on well-being and health. Residents were given a five-point Likert response format, with the following options: 1 = no effect at all (insignificant), 2 = minor negative effect, 3 = moderate negative effect, 4 = major negative effect, and 5 = severe (extreme) negative effect.
- The last part of the survey included two questions. Residents were asked if they would prefer to continue with the current continuous 24-hour work system or if they favor a change to 16-hour shifts where they are on-call from 4 p.m. to 8 a.m. the following day. Residents were also asked if their residency program has 24-hour consultant coverage for final reads.

### Statistical analysis

Statistical analysis was performed using Statistical Package for Social Sciences (IBM SPSS Statistics for Windows, Armonk, New York, USA), version 26. In order to assess the internal consistency of the survey, Cronbach’s α coefficient was calculated and was found to be > 0.7 (0.88). Descriptive statistics were performed for all variables. Categorical variables were expressed as frequency and percentage, while continuous variables were expressed as mean and standard deviation. To compare the means between males and females and the means between age groups (≤ 30 and > 30 years), Student’s t-test was performed. A one-way ANOVA test was performed to compare the means between different training levels. The significance level was set at P = 0.05. The relative importance index (RII), described by Holt et al.^15^, was used to rank the different indicators of the negative impact from the most important to the least important. Summative analysis was used to calculate the means and standard deviations for each item related to the perceived negative impact of the 24-hour work system. Pearson’s chi square test was used to assess the relationship between gender and perception of the negative impact of 24-hour shifts.

## RESULTS

Of the 337 eligible residents who received the invitation to participate, a total of 116 residents completed the survey during the study period, with a response rate of 34.4%. Males represented 55.2% of participants (n = 64), and the majority were ≤ 30 years old (n = 107; 92%). Most were single (n = 71; 61.2%). Of the participating residents, 34% (n = 40) were in their first year of radiology residency training (R1), and 16% (n = 19) were in their fourth year of training (R4).

Student’s t-test showed no significant difference in the mean of the perceived negative impact between males and females, between the two age groups (≤ 30 and > 30 years), or between those who have 24-hour consultant coverage and those with no consultant coverage (P > 0.05). The one-way ANOVA test also showed no significant difference between residents at different training levels or in different administrative regions of training. Demographic characteristics of participating residents and their relationship with the overall perceived negative impact of 24-hour shifts are shown in Table-I.

**Table I:** Demographic characteristics of participating residents and their relationship with the negative impact of 24-hour shifts

Variables	n (%)	Mean (SD)	Statistics	P-value
***Age***				
≤30	107 (92.2%)	3.6 (0.79)	t = -0.40	0.68
>30	9 (7.8%)	4.0 (0.87)		
***Gender***				
Male	64 (55.2%)	3.80 (0.77)	t = 1.36	0.83
Female	52 (44.8%)	3.60 (0.83)		
***Training level***				
R-1	40 (34.5%)	3.57 (0.82)	f = 1.33	0.26
R-2	28 (24.1%)	3.95 (0.75)		
R-3	29 (25.0%)	3.65 (0.77)		
R-4	19 (16.4%)	3.73 (0.84)		
***Region of training***				
Central	50 (43.1%)	3.71 (0.79)	f = 1.89	0.11
North	2 (1.7%)	3.18 (0.44)		
South	4 (3.4%)	3.93 (0.89)		
Western	43 (37.1%)	3.55 (0.82)		
Eastern	17 (14.7%)	4.12 (0.66)		
*Consultant coverage during duty for final reads (in-house or reading from home)*				
Yes	52	32.11 (6.04)	t = 0.95	0.34
No	64	33.20 (8.55)		

Ranking of indicators using the relative importance index: The negative impact on sleep rhythm during the post-call day ranked first (RII 0.84, mean 4.23 ± 1.01), followed by the impact on social life, family activities, and exercise during the post-call day (RII 0.81, mean 4.09 ± 1.06). The third highest ranking factor was missing academic activities on the post-call day (RII 0.78, mean 3.91 ± 1.15). The negative impact on overall well-being and health ranked at the bottom of the list (RII 0.65, mean 3.28 ± 1.12). Table-II shows the RII and rank of different items related to the negative impact of 24-hour duty.

**Table-II:** Relative importance index ranking the perceived negative impacts of 24-hour duty from highest to lowest.

Questions	Mean (SD)	RII	Rank
Sleep rhythm during the post-call day	4.23 (1.01)	0.84	1
Social life, family activities, and exercise during the post-call day	4.09 (1.06)	0.81	2
Missing academic activities on the post-call day	3.91 (1.15)	0.78	3
Mood and humor during the post-call day	3.86 (1.15)	0.77	4
Reduction in performance due to fatigue while on-call	3.57 (1.02)	0.71	5
Reduction in decision-making capability while on-call	3.38 (1.13)	0.67	6
Overall well-being and health	3.28 (1.12)	0.65	7

RII – relative importance index.

A total of 87.9% (102 of 116) of residents indicated their preference for 16-hour shifts, while only 12.1% (14 of 116) preferred to continue with the 24-hour duty system. A total of 44.8% (52 of 116) of residents indicated that they have 24-hour consultant coverage to finalize reports through an in-house consultant or a consultant reading and finalizing studies from home, while 55.2% (64 of 116) indicated that they do not have 24-hour consultant coverage. The student’s t-test showed no significant difference in the mean of the perceived negative impact between these groups.

### Impact of 24-hour duty based on gender

Table-III shows the relationship between the impacts of 24-hour duty and gender. No significant relationship (P > 0.05) was found between gender and the eight items concerning the perceived negative impact of night duty, the negative impact on the post-duty period, or the overall negative impact on well-being and health.

**Table-III:** Relationship between the negative impact of 24-hour duty and gender.

Questions		No effect	Minor negative effect	Moderate negative effect	Major negative effect	Severe negative effect	P value
Sleep rhythm during the post-call day	Male	0	2	9	11	42	0.085
	Female	2	4	10	14	22	
Social life, family activities, and exercise during the post-call day	Male	0	5	13	16	30	0.333
	Female	3	2	9	13	25	
Missing academic activities on the post-call day	Male	1	7	10	14	32	0.214
	Female	3	5	12	16	16	
Mood and humor during the post-call day	Male	4	5	11	17	27	0.654
	Female	2	4	12	18	16	
Reduction in performance due to fatigue while on-call	Male	0	10	21	17	16	0.320
	Female	2	5	18	18	9	
Reduction in decision-making capability while on-call	Male	3	7	24	15	15	0.711
	Female	5	7	19	13	8	
Overall well-being and health	Male	3	11	20	16	14	0.301
	Female	5	7	22	13	5	

## DISCUSSION

The aim of this study was to assess the perceived negative impact of long duty hours on education and personal well-being among medical trainees in the diagnostic radiology residency training program in Saudi Arabia. The results of this study are consistent with those previously reported in the literature, which associate long duty hours with various negative outcomes.^4^,^5^ The impact on sleep rhythm ranked highest in our participants. There is a noticeable increase in demand for radiology services after regular working hours, which may lead to long duty hours and ultimately sleep deprivation among radiology residents, as was evident in medical residents.^4^ The National Sleep Foundation recommends 7–9 hours of sleep per night.^16^ According to an experiment by Lamond et al.^17^, two recovery sleep periods of at least nine hours each after one night of sleep deprivation are needed to reverse the effect of sleep loss and restore both vigilance and performance. Insufficient hours of recovery sleep after duty may alter the circadian rhythm and cause disturbance in sleep, which can impact other aspects of work life balance.^18^,^19^

The impact on social life, family activities, and exercise ranked second. Residents’ overall health and attitude can have an important and direct impact on their relationships with their family.^20^ A previous study by Law et al.^21^ has described the negative effects of long duty hours on residents’ roles outside the hospital. Studies by Ahmed et al.^6^ and Landrigan et al.^7^ suggest that long working hours are essential for residents’ training; however, succeeding in their training should not heavily impact their social and family lives.

Missing academic activities after duty ranked third. Academic activities are an important and integral part of residents’ educational needs during residency training,^22^ helping residents to gain the competencies needed to enter practice. According to Wolpaw et al.^23^, educational needs must not be compromised by increased reliance on residents to fulfill duty obligations. Recovery sleep usually takes place after duty. Subsequently, the on-call resident will probably not be able to attend the academic activities scheduled on the day following duty. Missing academic activities after duty is also observed among residents in other specialties and was recently reported by Alsohime et al.^14^ among pediatric residents.

The impact on mood and humor ranked fourth. Factors affecting mood and humor may include burnout, anxiety, and depression.^24^ High rates of burnout were reported in one-fourth of radiology residents in Riyadh according to a recent study by Bin Dahmash et al.^25^ The association of burnout with long duty hours has also been reported in other healthcare residents.^26^,^27^ Depression and anxiety, on the other hand, are important factors that alter mood and need to be evaluated among residents using a validated tool. Currently, there are insufficient data in the literature regarding the prevalence of depression or anxiety among radiology residents in Saudi Arabia, and their relationship with long duty hours remains unclear.

Reduction in performance ranked fifth, while the reduction in decision-making capability ranked sixth. This is consistent with multiple studies in the literature on the cognitive and functional deficits in healthy adults who stay awake for 24 hours or more.^28^,^29^ This is also consistent with the Harvard Work Hours, Health, and Safety Study, which found that interns working in-hospital for 24 hours or more suffered increased attentional failures while working at night compared to those who worked 16 hours of scheduled consecutive work.^30^

The perceived negative impact on overall well-being and health ranked at the bottom of the list. Residents in training generally work more than 50 hours per week, which may have negative effects on their health and well-being. Working more than 50 hours per week is known to affect psychological, social, and physical well-being, placing workers at risk for sleep deprivation, fatigue, declines in alertness or concentration, and poorer general health.^31^,^32^ The maximum permitted number of duty hours varies considerably around the world. Decreasing duty hours is an ongoing process, and diverse approaches are already implemented by international jurisdictions in different countries.^33^ Different studies reported an improvement in residents’ overall well-being and sleep after restricting call duration, but these restrictions negatively impacted residents’ education and resulted in reduced supervision.^34^-^36^ Furthermore, there was no relationship between these negative impacts with gender. This was in contradiction to some of the previous studies on other healthcare providers, where female healthcare workers’ lives were more negatively impacted by long working hours.^37^,^38^

### Limitation of this study

The main limitation of this study is that it assessed and evaluated the subjective perception of the 24-hour call-duty system instead of an objective measurement. Variation in workload between different training centers during on-call duty was not taken into consideration. Other limitations include the cross-sectional design and possible self-reporting inaccuracies. Also, this study did not investigate the residents’ preference for the night float system. This system is not currently implemented by any training center in Saudi Arabia.

## CONCLUSION

In this study, we found that the 24-hour duty system negatively impacted radiology residents’ well-being and education. In order to promote residents’ well-being and prepare them to be resilient radiologists, collaboration between a multidisciplinary team of experts and the residency training program committee is needed to anticipate potential challenges of implementing additional changes to duty hours. Although reduction in duty hours may increase performance and decrease the overall perceived negative impact on residents, we cannot substantiate that such policies improve residents’ quality of life. More research in the field is required to develop evidence-based policies that promote residents’ well-being with minimal impact on their education.

### Authors’ Contribution:

**AA:** Conception, design, study performance, manuscript writing.

**SA:** Data collection and interpretation.

**YA:** Data collection and interpretation.

**TD:** Analysis of data and drafting and critically revising manuscript.

The corresponding author is responsible and accountable for the accuracy or integrity of the work.
